# 157 ’Finding Our Tribe’: Parental Experiences Navigating Sport and Physical Activity Opportunities for Neurodivergent Children and Young People

**DOI:** 10.1093/eurpub/ckae114.142

**Published:** 2024-09-26

**Authors:** Erin Byrd, Angela Carlin, Ben Fitzpatrick, Laura Hemmings

**Affiliations:** Ulster University, Derry, United Kingdom; Ulster University, Derry, United Kingdom; Ulster University, Derry, United Kingdom; University of Birmingham, Birmingham, United Kingdom

## Abstract

**Purpose:**

Neurodivergent children and young people (CYP) experience higher risk of poor physical and mental health. Many community programmes have been designed to provide young people with a physical activity (PA) outlet to improve health and wellness, however, neurodivergent CYP and their families continue to face stigmatising attitudes and exclusion when trying to access these opportunities. This study aimed to explore the experience of parents and caregivers of neurodivergent CYP, in the United Kingdom (UK), in finding, accessing, and engaging with inclusive community sport and physical activity opportunities and resources for their children.

**Methods:**

An online qualitative survey explored the experience of parents and caregivers of neurodivergent CYP in the UK in finding and accessing community sport and PA opportunities. One-hundred and twelve parents and caregivers completed the survey from across all 4 devolved nations in the UK. Recruitment of participants occurred through collaboration with parent and advocacy groups for neurodivergent populations, charities providing services to these populations across the UK, as well as through social media platforms. Data collected was interpreted using inductive-thematic analysis, allowing for emergent themes to be identified using a constructivist framework.

**Results:**

Qualitative data revealed an overarching theme: the ‘Constant Struggle’ parents of neurodivergent CYP face in trying to find, access, and support their children to participate in sport and physical activities. Four main themes emerged under this overarching theme including: 1) An awareness of inequity in the sport and physical opportunities available, 2) A need for flexibility and adaptability, 3) The importance of the coach, and 4) Empowering the parent through inclusion and support.

**Conclusion:**

Parents and caregivers of neurodivergent CYP express the desire for more equitable provision and delivery of community sport and PA opportunities. It is recognised that there are currently challenges in identifying and accessing appropriate opportunities, however, solutions are offered that will support parents to feel more empowered to engage with sport and PA opportunities.

**Support/Funding Source:**

Supported by Ulster University and the University of Birmingham.

